# Trends in Missing Race and Ethnicity Information After Imputation in HealthCare.gov Marketplace Enrollment Data, 2015-2021

**DOI:** 10.1001/jamanetworkopen.2022.16715

**Published:** 2022-06-10

**Authors:** D. Keith Branham, Kenneth Finegold, Lucy Chen, Melony Sorbero, Roald Euller, Marc N. Elliott, Benjamin D. Sommers

**Affiliations:** 1 US Department of Health and Human Services (HHS), Washington, DC; 2 Office of the Assistant Secretary for Planning and Evaluation (ASPE), Washington, DC; 3 Harvard Graduate School of Arts and Sciences, Cambridge, Massachusetts; 4 Havard Business School, Boston, Massachusetts; 5 RAND Corporation, Santa Monica, California

## Abstract

This cross-sectional study examines patterns of missing information on race and ethnicity after an imputation of HealthCare.gov enrollment data between 2015 and 2021.

## Introduction

Demographic characteristics of Marketplace enrollees can inform health equity policy and research. However, more than 30% of HealthCare.gov enrollees do not report race and ethnicity. This cross-sectional study uses imputation to describe HealthCare.gov enrollment data and assess patterns of missing enrollee race and ethnicity.

## Methods

This study was reviewed and approved by RAND Corporation’s institutional review board. Informed consent is not applicable to this study. This study followed the STROBE reporting guideline. We used open enrollment data from 2015 through 2021 for states using the HealthCare.gov platform from the Centers for Medicare and Medicaid Services’ Multidimensional Information and Data Analytics System (MIDAS). We categorized enrollees into 7 mutually exclusive, self-reported groups: American Indian or Alaska Native; Asian American, Native Hawaiian, and Pacific Islander; Black; Hispanic (regardless of race); multiracial; White; and missing. These categories were based on information available in MIDAS and were consistent with open enrollment reports.

For enrollees missing data in one year but not in another, we used the closest available plan year to fill in the missing year(s). We then used the modified bayesian improved first name surname geocoding (mBIFSG) method to generate probabilities for the 6 nonmissing categories for remaining missing values.^1^ This approach uses 6 million surnames from 2010 Census data and 4250 first names from 2.5 million mortgage applications in 2007 and 2010 to assign race and ethnicity probabilities and links an enrollee’s address to Census block group racial and ethnic composition.^2,3^ Finally, we calibrated the posterior probabilities to the observed distribution of self-reported race and ethnicity.

We evaluated results using C statistics,^4^ combined self-reported and imputed data, and assessed trends in Marketplace enrollment by race and ethnicity. Estimates used 6 imputed probabilities summing to 1 for each enrollee rather than assigning race or ethnicity to enrollees.^5^ Analyses were conduced using SAS, version 9.4 (SAS Institute).

## Results

From 2015 to 2021, 24.4 million unique enrollees selected 61.4 million plans. Race and ethnicity was missing in 31.5% of plans (22.2% after using other plan-year data; <0.01% after imputation). Imputation had an overall C statistic of 0.94, ranging from 0.62 to 0.97 across categories. Discernment was very high (C statistic >0.90) for Asian American, Native Hawaiian, and Pacific Islander; Black; Hispanic; and White but lower for American Indian or Alaska Native (C statistic = 0.62) and multiracial (C statistic = 0.67).

Annual percent missing ranged from 26.3% to 36.1% (Figure); imputation results indicated nonreporters were disproportionately Black or Hispanic. Estimated distribution of enrollment by category, before and after imputation (Figure, A vs B), for 2021 changed from 6.3% to 9.5% for Asian American, Native Hawaiian, and Pacific Islander; 6.2% to 11.3% for Black; 12.5% to 22.6% for Hispanic; and 38.7% to 54.0% for White.

**Figure.**
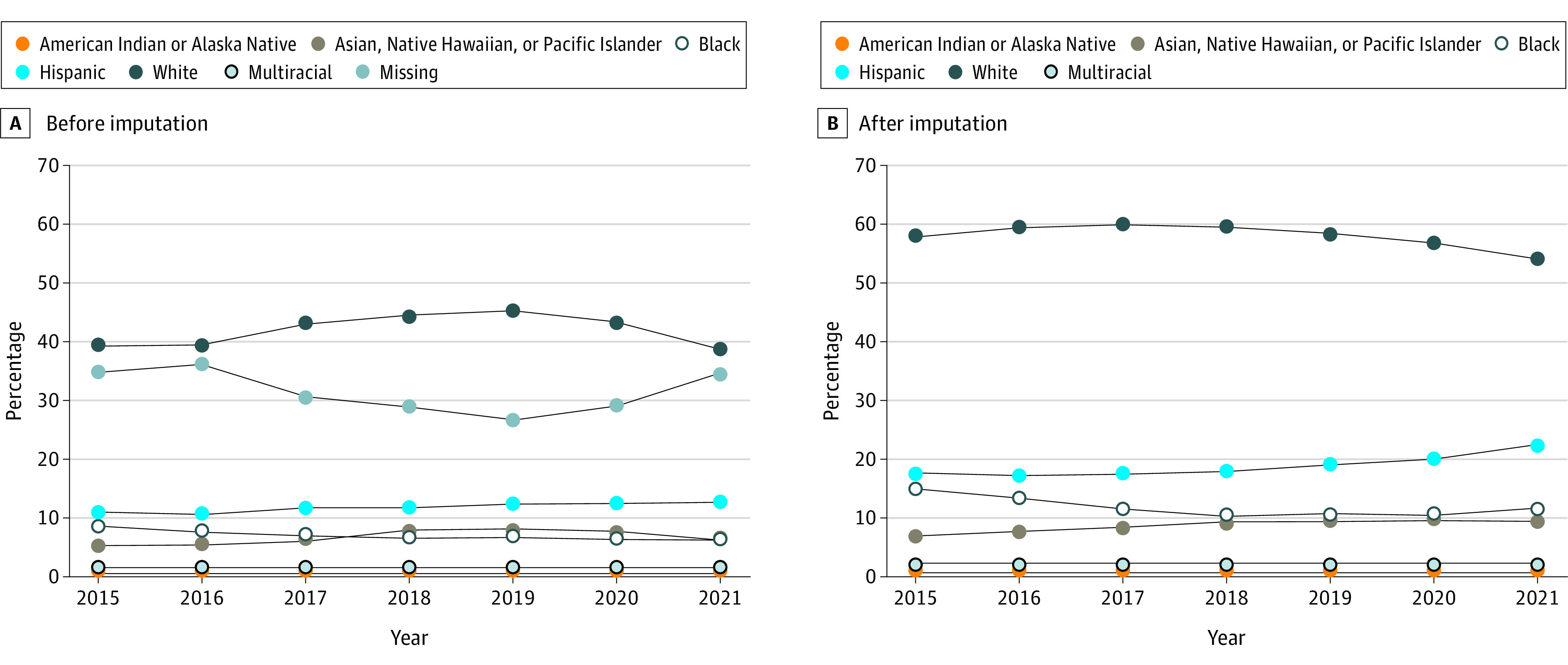
Trend in Federal Marketplace (HealthCare.gov) Enrollees’ Race and Ethnicity Before and After Imputation, 2015-2021 The distribution of race and ethnicity are shown before (A) and after (B) imputation of race and ethnicity, by year, for federal Marketplace (HealthCare.gov) enrollees. The method uses name, address, and Census block group information to estimate the probabilities of race and ethnicity categories for enrollees with missing values.

## Discussion

Imputation of race and ethnicity in HealthCare.gov data using the mBIFSG imputation method had high overall predictive accuracy except for American Indian or Alaska Native and multiracial categories. Imputed results indicate that enrollees with missing data were disproportionately Black and Hispanic and that the Marketplace may serve a more diverse population than suggested by self-reported data.

Imputation of race and ethnicity may improve researchers’ and policy makers’ ability to examine disparities and design better outreach and enrollment strategies. Imputation of race and ethnicity is common in Medicare data^6^; here, we demonstrate its potential use in Marketplace data. Nonetheless, policy efforts should aim to improve self-reported race and ethnicity data collection.

Limitations include the imputation assumption that nonreporters are otherwise similar to self-reporters with the same names and Census block group, the use of older Census data owing to unavailability of 2020 data during the analysis, and the potential that mortgage application data may misrepresent racial and ethnic groups because of longstanding structural inequities in housing. A Census first-name list and additional Marketplace data could further improve imputation. Future research may use imputation to understand consumer experiences in the Marketplace and improve outreach effectiveness to address racial and ethnic inequity.
